# P21 Humidity and moisture exchange filters: assessment of a novel microbiological sampling technique for patients with ventilator-associated pneumonia

**DOI:** 10.1093/jacamr/dlaf230.028

**Published:** 2025-12-04

**Authors:** Samuel Quarton, Nicola Cumley, Mahboobeh Behruznia, Dhruv Parekh, Alan McNally, Elizabeth Sapey, David Thickett

**Affiliations:** NIHR Birmingham Biomedical Research Centre, Institute of Translational Medicine, Birmingham B15 2TH, UK; NIHR Birmingham Biomedical Research Centre, Institute of Translational Medicine, Birmingham B15 2TH, UK; NIHR Birmingham Biomedical Research Centre, Institute of Translational Medicine, Birmingham B15 2TH, UK; NIHR Birmingham Biomedical Research Centre, Institute of Translational Medicine, Birmingham B15 2TH, UK; NIHR Birmingham Biomedical Research Centre, Institute of Translational Medicine, Birmingham B15 2TH, UK; NIHR Birmingham Biomedical Research Centre, Institute of Translational Medicine, Birmingham B15 2TH, UK; NIHR Birmingham Biomedical Research Centre, Institute of Translational Medicine, Birmingham B15 2TH, UK

## Abstract

**Background:**

Ventilator-associated pneumonia (VAP) is a common, high-consequence complication of mechanical ventilation. High rates of antimicrobial resistance in VAP and varied potential pathogens necessitate widespread broad-spectrum empirical choices, which in turn drives selection pressure and further antimicrobial resistance. Invasive sampling with bronchoalveolar lavage can identify organisms to guide treatment, however this carries risks and may not be suitable for all patients. As such, non-invasive sampling techniques could improve rates of pathogen identification and enable more targeted antimicrobial treatment. Heat and moisture exchange (HME) filters within the ventilator circuit offer an exciting potential sampling site that would be applicable for all patients with suspected VAP.

**Objectives:**

To explore the utility of sampling the humidity and moisture exchange (HME) filter within the ventilator circuit as a non-invasive method of pathogen identification.

**Methods:**

Patients not treated for infection were recruited within 48 h of intubation. HME filters were collected after 24 h in-situ, together with concurrent non-directed bronchoalveolar lavage (NDBAL). Samples were repeated after 3–5 days and 7–10 days, or at development of pneumonia (based on clinical diagnosis). HME filters were swabbed and cfu/mL from quantitative culture compared to that of NDBAL, alongside metagenomic sequencing of samples. Organisms identified and antibiotics received were reviewed.

**Results:**

Fifteen patients were recruited, of whom seven developed pneumonia. In total 31 NDBAL were performed and 33 HME filters collected. On three occasions, NDBAL was not possible due to patients’ clinical status, highlighting potential benefits of a non-invasive technique. Overall sensitivity for identifying organisms was low for both NDBAL and HME filters (55% and 42% respectively), but better in samples taken within 48hrs of pneumonia diagnosis (NBAL 71%, HME 57%). Growth from the HME was more specific for pneumonia than NDBAL (83% versus 45%). Pathogens identified from HME filters showed reasonable concordance with NDBAL and included fastidious bacteria such as *Haemophilus influenzae.* Four cases matched completely, one was a partial match and two were completely discordant. Eighteen time points were negative by both methods.

**Conclusions:**

HME filters show promise as a readily available sampling site for pathogen identification in VAP. While sensitivity is lower than NDBAL, specificity for infection appears high. Given the low cost involved, there may therefore be potential for including swabs of HME filters within screening tools for VAP. Further work may identify host or pathogen-specific factors that impact its utility, and assessment is ongoing.

